# Economy in the Generation and Use of Steam

**Published:** 1914-05-30

**Authors:** 


### ECONOMY IN THE GENERATION AND USE OF STEAM.

### I. Sources of Waste in the Older Boilers.

Steam is now used in hospitals for a large number of purposes. It is used to a small extent for power—chiefly electric lighting and motive power. It is used to a large extent for heating water for the various purposes required about a hospital, including the heating of the wards.

Steam is a very convenient agent for delivering heat. A comparatively large quantity of heat can be stored in a small quantity of steam, and the steam will deliver up the heat to any agent, such as water, that is arranged to receive it. The question of economy in generating steam, and in using it, has been closely studied of late. Steam engineers have had to face the keen competition of gas and oil, and they have worked out a number of appliances, all designed to prevent the waste of the heat that took place previously. In the generation of steam the temperature of water must be raised from the normal, say, 60° F. or 70° F., sometimes lower in winter, to boiling temperature (212° F.). In order to do this a certain definite number of thermal units have to be delivered to the water. The British thermal unit, which is the standard all over the civilised world, is the quantity of heat required to raise the temperature of 1 lb. of water from 39° F. to 40° F. For practical purposes it is usually taken to be the quantity of heat required to raise the temperature of 1 lb. of water 1° anywhere in the Fahrenheit scale. When water reaches boiling-point a very large expenditure of heat is required to convert it into steam. While, for instance, 152 B.Th. units are required to raise the temperature of 1 lb. of water from 60° F. to 212° F., 966 B.Th. units have to be delivered to convert it into steam at 212° F. For hospital purposes steam is generated at a pressure considerably above that of the atmosphere; the result is that instead of 966 B.Th. units, only 897 units are required if the steam is under 60 lb. pressure.

In the process of generating steam, fuel of various kinds, coal usually in hospitals, is burnt in a furnace, and a large volume of hot gases, formed from the combustion of the coal, is caused to circulate over the metal surfaces of the boiler. Several sources of waste arise here. The fuel may be burnt wastefully. A bad stoker may use much more coal than a good stoker would, and will usually maintain a very uneven steam-pressure. In order to keep the furnace fed with coal the furnace doors have to be opened periodically, and on every such occasion a draught of cold air enters the furnace and interferes with the combustion. In modern boiler plant the coal is fed automatically into the furnace (by apparatus to be described in the next article, known as a mechanical stoker), and the furnace doors are rarely opened. This has the double effect of economising whatever fuel may be used, and it also enables a much lower grade of fuel to perform the same work. Previously to the introduction of the mechanical stoker it was economical to buy the best coal; now it is economical to buy almost the worst.

In addition, the mechanical stoker enables a very much thicker and hotter fire to be carried in the furnace, providing that another aid, known as mechanical draught, is applied. In most hospitals it would be a great boon to be rid of the tall chimney. Mechanical draught, which is produced by the aid of a fan driven by an electric motor, gives a much better result in every way than a chimney: it is more economical, and it is more under control.

In addition to this, where a chimney is depended upon to create the draught a large proportion of the heat that is liberated in the furnace is required to create the draught in the chimney. The hot gases are delivered to the chimney at a temperature of 600° F. and upwards. With mechanical draught they can be delivered at 300° F. and downwards; and the difference between 600° F. and 300° F. is utilised in heating the water with which the boiler is to be fed.

Another source of waste in the older forms of steam boiler arose from the want of circulation of the water in the boiler itself, and from the delivery to the boiler of comparatively cold water. By the aid of the "Economiser," to be described in the next article, the hitherto wasted heat of the hot flue gases is employed, and the hitherto wasted heat of the exhaust steam raises the water with which the boiler is fed to the same temperature as that which is already in the boiler. There is a double saving by this. The heat units required to raise the temperature of the water from 60° to that of the steam in the boiler is saved, and the check to the circulation caused by the cold feed-water is also avoided.

